# Association Between Kidney Function Decline and Baseline TNFR Levels or Change Ratio in TNFR by Febuxostat Chiefly in Non-diabetic CKD Patients With Asymptomatic Hyperuricemia

**DOI:** 10.3389/fmed.2021.634932

**Published:** 2021-07-12

**Authors:** Tomohito Gohda, Naotake Yanagisawa, Maki Murakoshi, Seiji Ueda, Yuji Nishizaki, Shuko Nojiri, Yasuo Ohashi, Iwao Ohno, Yugo Shibagaki, Naohiko Imai, Satoshi Iimuro, Masanari Kuwabara, Hiroshi Hayakawa, Kenjiro Kimura, Tatsuo Hosoya, Yusuke Suzuki

**Affiliations:** ^1^Department of Nephrology, Juntendo University Faculty of Medicine, Tokyo, Japan; ^2^Medical Technology Innovation Center, Juntendo University, Tokyo, Japan; ^3^Department of Integrated Science and Engineering for Sustainable Society, Chuo University, Tokyo, Japan; ^4^Division of General Medicine, Department of Internal Medicine, The Jikei University School of Medicine, Tokyo, Japan; ^5^Division of Nephrology and Hypertension, Department of Internal Medicine, St. Marianna University School of Medicine, Kawasaki, Japan; ^6^Innovation and Research Support Center, International University of Health and Welfare, Tokyo, Japan; ^7^Intensive Care Unit, Department of Cardiology, Toranomon Hospital, Tokyo, Japan; ^8^Internal Medicine, Kanto Central Hospital, Tokyo, Japan; ^9^Tokyo Takanawa Hospital, Tokyo, Japan; ^10^Division of Chronic Kidney Disease Therapeutics, The Jikei University, Tokyo, Japan

**Keywords:** TNF receptor, CKD, uric acid, xanthine oxidase inhibitor, eGFR

## Abstract

**Background:** The levels of circulating tumor necrosis factor receptor (TNFR) 1 and 2 help predict the future decline of estimated glomerular filtration rate (eGFR) chiefly in patients with diabetes. It has been recently reported that the change ratio in TNFR1 by SGLT2 inhibitor treatment is also related with future GFR decline in patients with diabetes. The aims of this study are to investigate the association between baseline TNFR levels and early change in TNFR levels by the non-purine selective xanthine oxidase inhibitor, febuxostat, and future eGFR decline chiefly in chronic kidney disease (CKD) patients without diabetes.

**Methods:** We conducted a *post-hoc* analysis of the FEATHER study on patients with asymptomatic hyperuricemia and CKD stage 3, who were randomly assigned febuxostat 40 mg/day or matched placebo. This analysis included 426 patients in whom baseline stored samples were available. Serum TNFR levels at baseline were measured using enzyme-linked immunosorbent assay. Those levels were also measured using 12-week stored samples from 197 randomly selected patients.

**Results:** Compared with placebo, short-term febuxostat treatment significantly decreased the median percent change from baseline in serum uric acid (−45.05, 95% CI −48.90 to −41.24 mg/dL), TNFR1 (1.10, 95% CI−2.25 to 4.40), and TNFR2 (1.66, 95% CI −1.72 to 4.93), but not TNFR levels. Over a median follow-up of 105 weeks, 30 patients (7.0%) experienced 30% eGFR decline from baseline. In the Cox multivariate model, high levels of baseline TNFR predicted a 30% eGFR decline, even after adjusting for age, sex, systolic blood pressure, high sensitivity C-reactive protein, uric acid, and presence or absence of febuxostat treatment and diabetes, in addition to baseline albumin to creatinine ratio and eGFR.

**Conclusion:** Early change in circulating TNFR levels failed to predict future eGFR decline; however, regardless of febuxostat treatment, the elevated baseline level of TNFR was a strong predictor of 30% eGFR decline even in chiefly non-diabetic CKD patients with asymptomatic hyperuricemia.

## Introduction

Hyperuricemia was previously considered to occur because of reduced renal clearance of uric acid in patients with decreased renal function; therefore, treatment of asymptomatic hyperuricemia has received less attention from researchers. However, several recent epidemiological studies demonstrated that the high levels of serum uric acid increased the risk of renal function decline in the general population (1, 2). In patients with type 1 diabetes, high-normal serum uric acid was reported to predict estimated future decline in glomerular filtration rate (eGFR) (3). However, recent randomized controlled clinical trials (RCTs) demonstrated that the reduction of serum uric acid with allopurinol did not prevent renal function decline in patients with type 1 diabetes and mild to moderate diabetic kidney disease (DKD) or stage 3 and 4 chronic kidney disease (CKD), both of which are high risk factors for eGFR decline (4, 5). Data on halting the progression of CKD using non-purine selective xanthine oxidase inhibitor to lower uric acid have been insufficient (6, 7), although a number of studies demonstrated that uric acid was associated with the pathogenesis of kidney injury through endothelial dysfunction and inflammation (8, 9).

Studies indicated that the elevated levels of circulating tumor necrosis factor receptor (TNFR), which is one of the inflammatory markers, predicted the progression of renal function decline and mortality in a broad range of diseases (10–13). However, the mechanisms that modulate TNFR levels are not fully understood, although the *post-hoc* analyses of several clinical trials reported that several therapeutic agents altered serum TNFR levels (14, 15). We and other researchers previously demonstrated that serum TNFR level was associated with serum uric acid level in patients with type 2 diabetes (16, 17).

Therefore, in the current *post-hoc* analysis of the previously reported FEATHER study, we investigated whether baseline TNFR levels and early change in circulating TNFR levels predicted 30% eGFR decline chiefly in non-diabetic CKD patients with asymptomatic hyperuricemia.

## Methods

### Study Patients

The FEATHER trial design, baseline characteristics of the participants, and primary results have been previously described (6). This was a *post-hoc* analysis of a multicenter, prospective, double-blind, randomized, placebo-controlled parallel group trial to test and compare the efficacy of the xanthine oxidase inhibitor (febuxostat) with placebo in preventing the progression of CKD. Briefly, in this study, 467 patients aged ≥ 20 years, in CKD stage 3 (eGFR 30–60 mL/min/1.73 m^2^) with asymptomatic hyperuricemia were eligible for enrollment. Moreover, participants were required to have a uric acid concentration between 7.0 and 10.0 mg/dL. The study patients were randomized to receive febuxostat 40 mg/day or placebo for up to 108 weeks. In this study, we included 426 patients whose baseline samples were available.

### Measurement of Circulating TNFR Concentrations

Direct sandwich enzyme-linked immunosorbent assay was used for quantifying TNFR1 and TNFR2 (R&D systems, Minneapolis, Minnesota), as previously described (18, 19). To assess the effect of febuxostat, we measured serum TNFR levels using baseline and 12-week stored samples from 197 randomly selected patients. Moreover, the baseline TNFR levels were measured in 426 patients to examine whether they predicted renal function decline.

### Statistical Analysis

Data were expressed as mean ± standard deviation (SD) or as median (25th−75th percentile). Categorical variables were then assessed using the chi-square test. Moreover, baseline demographics and clinical characteristics were assessed according to the baseline TNFR level. The Wilcoxon rank-sum test was also applied for comparison between–two groups.

Percentages of change in uric acid, TNFR1, and TNFR2 over the intervention period from baseline to 12 weeks were assessed for the febuxostat and placebo groups. Moreover, we calculated percentages of change in the albumin to creatinine ratio (ACR) and eGFR from baseline to end of study. The effect of febuxostat compared to placebo on circulating ACR, eGFR, uric acid, and TNFR levels was determined by the Wilcoxon rank-sum test. The median differences between groups and 95% CIs were estimated with the Hodges–Lehmann method. Cumulative incidence of 30% eGFR decline was estimated using Kaplan–Meier curves. Univariate and multivariate Cox proportional hazard regression analyses were applied to analyze the association between baseline TNFR levels and ≥30% eGFR decline, with adjustments for age, sex, presence of diabetes, systolic blood pressure (BP), high sensitivity C-reactive protein (hsCRP), ACR, eGFR, uric acid, and febuxostat treatment at baseline. The slope of eGFR (mL/min/1.73 m^2^/year) was determined using a random coefficient model in which the regression coefficients (intercept and slope) were assumed to be a random effect. In this model, an unstructured covariance matrix was specified for random intercept and slope effects, and all measurements were used to compute regression slopes; i.e., at baseline, at 4, 8, and 12 weeks, and then every 12 weeks until week 108 or until discontinuation of treatment. Moreover, multivariate linear regression analysis was performed to evaluate the independent association between TNFR levels and the slope of eGFR change, with adjustments for age, sex, presence of diabetes, systolic BP, hsCRP, ACR, eGFR, uric acid, and febuxostat treatment at baseline.

Statistical analyses were performed with SAS software version 9.3 (SAS Institute, Cary, NC, USA). A *P*-value of <0.05 was considered to be statistically significant.

## Results

### Characteristics of the Study Patients According to the Baseline TNFR Level

Table 1 shows the demographic and baseline characteristics. Compared with patients in the lowest tertile of TNFR2 level, those in the highest tertile of TNFR2 level comprised fewer men; had higher systolic BP, uric acid, and ACR; and had lower eGFR at baseline. However, age, body mass index (BMI), diastolic BP, glycated hemoglobin (HbA1c), hsCRP, presence of diabetes, and febuxostat treatment did not differ between both groups.

**Table 1:** Characteristics of the study patients according to the circulating TNFR2 levels.

**Variables**	**All**	**T1 + T2**	**T3**	*p*-value*
	**(*n* = 426)**	**(*n* = 284)**	**(*n* = 142)**	
TNFR1, pg/mL	2039 (1,585, 2,513)	1,733 (1,480, 2,094)	2,776 (2,395, 3,171)	<0.001
TNFR2, pg/mL	4,194 (3,339, 5,228)	3,582 (2,993, 4,194)	5,662 (5,228, 6,474)	<0.001
Febuxostat treatment, *n* (%)	214 (50.2)	146 (51.4)	68 (47.9)	0.493
Age, yr	65.4 ± 12.0	64.6 ± 11.5	66.9 ± 12.8	0.062
Male, *n* (%)	331 (77.7)	235 (82.8)	96 (67.6)	<0.001
Presence of diabetes, *n* (%)	131 (30.8)	85 (29.9)	46 (32.4)	0.603
BMI, kg/m^2^	24.8 ± 4.0	24.8 ± 3.8	24.8 ± 4.5	0.881
Systolic BP, mmHg	131.1 ± 15.1	130.0 ± 14.4	133.1 ± 16.2	0.046
Diastolic BP, mmHg	77.5 ± 11.1	77.9 ± 10.7	76.8 ± 12.0	0.346
Uric acid, mg/dL	7.8 ± 0.9	7.7 ± 0.8	8.0 ± 1.1	<0.001
HbA1c (%)	5.9 (5.6, 6.3)	5.9 (5.6, 6.3)	5.9 (5.6, 6.5)	0.299
HsCRP, ng/mL	590 (237, 1,400)	567 (242, 1,190)	660 (229, 2,080)	0.085
ACR, mg/gCr	118 (17, 534)	61 (11, 394)	372 (78, 735)	<0.001
eGFR, mL/min/1.73 m^2^	45 ± 10	48 ± 9	39 ± 8	<0.001

*Data are presented as mean ± SD, median (quartiles) or as %*.

T1–T3, tertiles 1–3. *T1 + T2 vs. T3. Tertile boundaries are as follows. TNFR1 (pg/mL): 1,728 for the 33.3th and 2,326 for the 66.6th percentiles; TNFR2 (pg/mL): 3,582 for the 33.3th and 4,785 for the 66.6th percentiles.

*ACR, ratio of urinary albumin to creatinine; BMI, body mass index; BP, blood pressure; eGFR, estimated glomerular filtration rate; HbA1c, glycated hemoglobin; HsCRP, high sensitivity C-reactive protein; SD, standard deviation; TNFR, tumor necrosis factor receptor*.

### Effect of Febuxostat on Uric Acid, TNFR1, and TNFR2 Levels

The characteristics of the 197 randomly selected patients in the febuxostat and placebo groups are shown in Supplementary Table 1. Compared with patients on placebo treatment at week 12, those on febuxostat treatment had a more pronounced reduction in median uric acid level (−45.05, 95% CI −48.90 to −41.24 mg/dL) but had similar serum TNFR levels (Table 2).

**Table 2:** Percent changes in uric acid, TNFR1, and TNFR2 from baseline to week 12.

**Variables**	**Placebo**	**Febuxostat**	**Difference**	***p*-value**
	**(*n* = 98)**	**(*n* = 99)**	**(95% CI)**	
ACR, mg/gCr^*^	13.79 (−24.77 93.50)	8.70 (−37.07, 76.68)	−6.17 (−30.14, 15.60)	0.575
eGFR, mL/min/1.73 m^2^	0.00 (−5.56, 4.69)	−1.49 (−6.98, 6.67)	−0.13 (−2.63, 2.38)	0.876
Uric acid, mg/dL	−1.33 (−6.49, 4.35)	−46.24 (−57.35, −35.00)	−45.05 (−48.90, −41.24)	<0.001
TNFR1, pg/mL	1.89 (−5.29, 9.43)	3.51 (−3.89, 10.90)	1.10 (−2.25, 4.40)	0.550
TNFR2, pg/mL	1.55 (−6.51, 7.22)	3.42 (−6.08, 10.14)	1.66 (−1.72, 4.93)	0.338

*Abbreviations used in this table are the same as in Table 1*.

*Data are presented as median (quartiles)*.

*The median differences between groups and 95% CI were estimated with the Hodges–Lehmann method*.

*P-values were computed from the Wilcoson rank-sum test*.

* *Percent change in ACR from baseline to end of study*.

### Effect of TNFR Levels on Future eGFR Decline: Univariate and Multivariate Cox Proportional Hazards Regression Analyses

The mean eGFR slope significantly differed between patients with TNFR2 level in the highest tertile (−1.24 ± 2.21 mL/min/1.73 m^2^ per year) and those in the two lowest tertile (0.20 ± 1.91 mL/min/1.73 m^2^ per year) groups (difference, −1.43; 95% CI, −1.84 to −1.03; *P* < 0.001). A similar result was observed according to TNFR1 level (difference, −1.54; 95% CI, −1.95 to −1.14; *P* < 0.001) (Supplementary Table 2).

**Figure 1:**
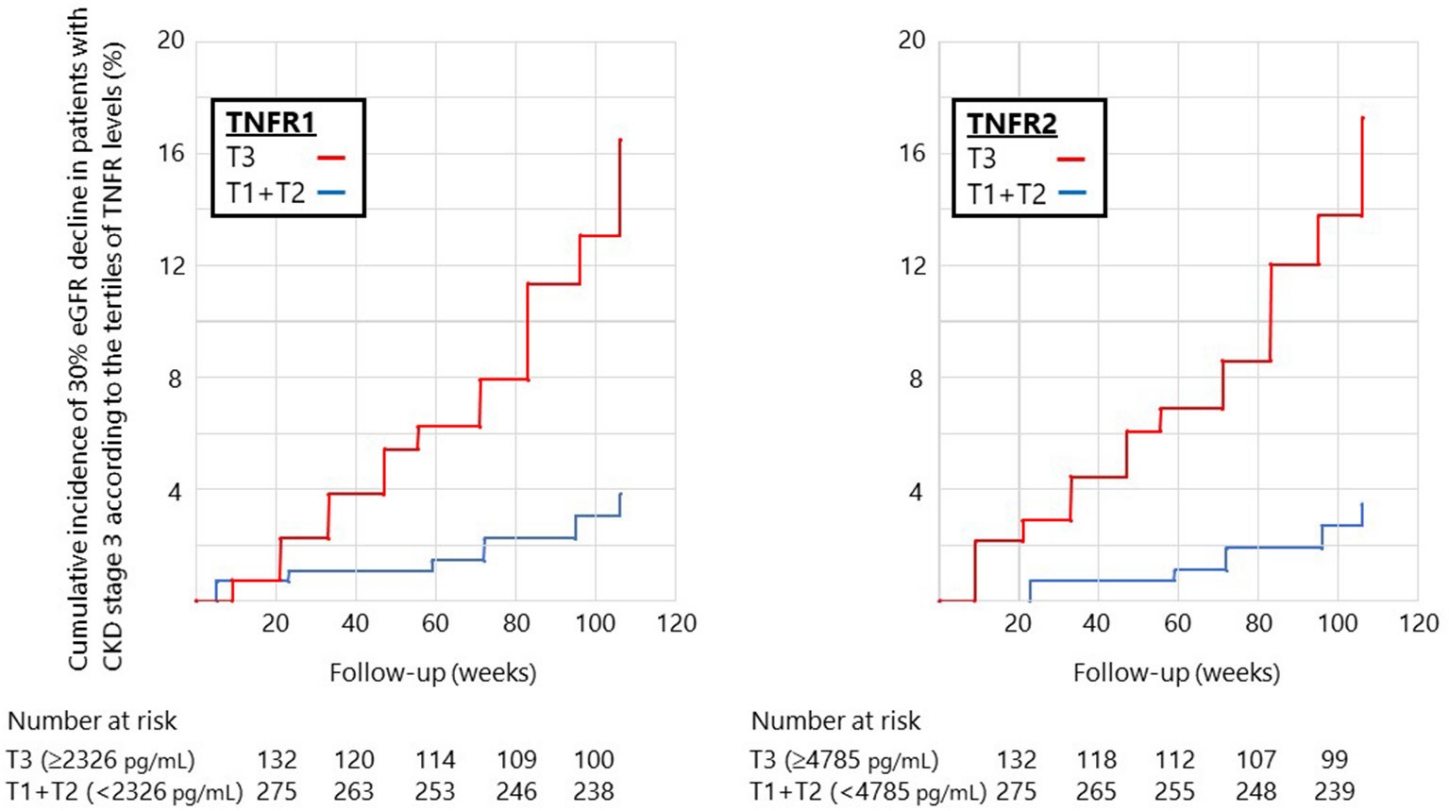
Cumulative incidence of 30% eGFR decline in patients with stage 3 CKD according to the tertiles of circulating TNFR levels at baseline. The cumulative incidence of 30% eGFR decline steeply increased at a constant rate from the start of observation for patients in the highest tertile of TNFR1 (left) or TNFR2 (right).

During the median follow-up period of 105 weeks, 12 (2.8%) and 30 (7.0%) patients developed the 40 and 30% eGFR decline, respectively. Therefore, we investigated the impact of circulating TNFRs on renal disease progression (i.e., 30% eGFR decline from baseline) using Cox proportional hazards regression model. In the univariate Cox regression model, high levels of TNFR2 and ACR at baseline significantly increased the incidence for 30% eGFR decline (Table 3). The cumulative incidence of 30% eGFR decline for the highest tertile of TNFRs steeply increased from start of observation (Figure 1). Overall, the risk of renal progression significantly increased in patients in the highest tertile of TNFR2 than in those in lowest two tertiles of TNFR2, after adjustment for age, sex, presence of diabetes, systolic BP, hsCRP, febuxostat treatment, ACR, and eGFR (HR 4.76, 95% CI 1.79–12.64, *P* = 0.002) (Table 3). Similar results were obtained when TNFR1 was included instead of TNFR2 in the model (Supplementary Table 3).

**Table 3:** Univariate and multivariate Cox regression analyses of the risk factors for 30% eGFR decline from baseline.

**Variables**	**Unadjusted**	***p*-value**	**Adjusted**	***p*-value**
	**HR (95%CI)**		**HR (95%CI)**	
TNFR2 (T3 vs. T1 + T2)	5.68 (2.50, 12.89)	<0.001	4.76 (1.79, 12.64)	0.002
Age (per 1 increase)	1.00 (0.97, 1.03)	0.908	1.00 (0.96, 1.03)	0.812
Male	1.21 (0.49, 3.00)	0.677	2.09 (0.77, 5.67)	0.150
Systolic BP (per 1 increase)	1.00(0.98, 1.03)	0.901	0.99 (0.96, 1.02)	0.388
Presence of diabetes	1.06 (0.48, 2.33)	0.892	0.85 (0.37, 1.95)	0.701
Treatment (Febuxostat vs. Placebo)	1.22 (0.59, 2.53)	0.601	1.35 (0.65, 2.84)	0.422
Uric acid (per 1 increase)	0.93 (0.62, 1.42)	0.746	0.76 (0.50, 1.14)	0.180
log_hsCRP (per 1 increase)	1.25 (0.95, 1.63)	0.108	1.18 (0.90, 1.55)	0.226
eGFR (per 1 increase)	0.96 (0.92, 1.00)	0.072	0.99 (0.95, 1.04)	0.789
log_ACR (per 1 increase)	1.42 (1.14, 1.78)	0.002	1.31 (1.02, 1.69)	0.034

*Abbreviations used in this table are the same as in Table 1*.

*log_, log-transformed*.

Next, multivariate regression model was used to examine the factors associated with the slope of eGFR rather than 30% eGFR decline. After adjusting all relevant variables in the multivariate regression model, baseline TNFR2, in addition to baseline eGFR and ACR, was significantly associated with the eGFR slope (Table 4). Moreover, similar results were obtained when TNFR1 was included rather than TNFR2 in the model (Supplementary Table 4).

**Table 4:** Multivariate regression analysis of the factors associated with eGFR slope in the study patients.

**Variables**	**Unstandardized coefficient**		**Standardized coefficient**	***p*-values**
	**B**	**SE**	**Beta**	
TNFR2 (T3 vs. T1, T2)	−0.547	0.233	−0.121	0.019
Age	0.000	0.009	−0.002	0.963
Male sex	−0.130	0.232	−0.026	0.575
Systolic BP	0.000	0.006	0.004	0.938
Presence of diabetes	0.148	0.207	0.032	0.477
Febuxostat treatment	0.249	0.183	0.059	0.175
Uric acid	0.051	0.104	0.022	0.627
log_hsCRP	0.058	0.073	0.035	0.423
eGFR	0.049	0.011	0.225	<0.001
log_ACR	−0.335	0.051	−0.316	<0.001

*Abbreviations used in this table are the same as in Table 1*.

*log_, log-transformed*.

## Discussion

In this *post-hoc* analysis, we tested the hypothesis that treatment with the xanthine oxidase inhibitor (febuxostat) would lower serum TNFR levels, and early change in circulating TNFR levels predicts future eGFR decline in patients with CKD stage 3. Our hypothesis was derived from the subsequent results of previous clinical and experimental studies: (1) uric acid stimulated renal inherent cells, such as mesangial cell, tubular cell, and endothelial cells, to produce inflammatory cytokines (20–23); (2) xanthine oxidase inhibitor treatment decreased uric acid level, resulting in attenuating macrophage infiltration to the kidney in diabetic rat (24); and (3) there was positive correlation between serum uric acid and circulating TNFR levels in patients with IgA nephropathy and diabetes (16, 17). In this study, serum TNFR level was not altered after 12 weeks of febuxostat treatment, although the baseline circulating TNFR level predicted the risk for 30% eGFR decline and was associated with eGFR slope, even after adjustment for relevant factors, including baseline eGFR and ACR.

A number of studies have shown the association of uric acid with inflammation, oxidative stress, and endothelial dysfunction in experimental models (20, 24, 25). Recently, Zhou et al. (26) showed that, compared with healthy individuals, even young patients with asymptomatic hyperuricemia had significantly higher inflammation and oxidative stress. Chronic inflammation plays a critical role in the development and/or progression of CKD. In human kidney biopsy samples, independent of crystal deposition, hyperuricemia was shown to be associated with tubular atrophy and interstitial inflammation and fibrosis (27). In animal models of several kidney diseases, hyperuricemia was demonstrated to induce macrophage infiltration, which was alleviated by treatment with xanthine oxidase inhibitor (24, 28). Although several studies indicated that treatment with allopurinol attenuated the increase in intercellular adhesion molecule-1 (ICAM-1) levels after acute ischemic stroke or decreased hsCRP in asymptomatic hyperuricemia (29, 30), recent placebo-controlled RCTs demonstrated that treatment with allopurinol did not improve endothelial function, oxidative stress, and inflammation in patients with stage 3 CKD (31). We previously demonstrated that serum uric acid is negatively associated with eGFR and positively associated with TNF-related markers such as TNFα and TNFRs in patients with IgA nephropathy and type 2 diabetes (16, 17). To date, therapeutic agents that modulate TNFR levels remain to be completely elucidated; however, several studies have revealed that treatment with sodium glucose cotransporter (SGLT2) inhibitor or Janus kinase (JAK) 1/2 inhibitor may decrease serum TNFR levels in patients with early and advanced DKD, respectively, whereas renin–angiotensin system inhibitor treatment did not alter TNFR levels in patients with early DKD (15). In this study, there were no differences in the TNFR levels between the intervention and non-intervention group.

Many observational studies demonstrated that high levels of uric acid predicted eGFR decline in patients with diabetes, CKD, and the general population (1–3). However, the FEATHER study reported that urate-lowering therapy failed to prevent renal function decline in patients with hyperuricemia and stage 3 CKD (6). One of the limitations of this study is that the eGFR decline in patients who received placebo is less (−0.47 ± 4.48 mL/min/1.73 m^2^), making it difficult to confirm the effect on renal function. Therefore, if we restrict the study patients whose baseline TNFR levels are high (i.e., patients whose eGFR steeply declines), the urate-lowering therapy may prevent renal function decline. However, in this study, use of febuxostat fails to prove this hypothesis (Supplementary Table 5).

To date, a growing body of evidence, including our research, demonstrated that circulating TNFR levels predict future eGFR decline chiefly in patients with diabetes. Similar to previous studies, our study expanded the prognostication value of TNFR on the renal function decline in part in non-diabetic population (32, 33). It is important to consider whether elevated circulating TNFR levels only reflect impaired renal processing of these proteins. Indeed, these levels are elevated in patients with advanced renal failure or hemodialysis patients (12, 13, 34). We demonstrated that increased TNFR production of any tissue, including kidneys, partly contributes to the elevation of those levels considering the measurements of fractional excretion TNFR (35). On the other hand, Niewczas et al. (15) demonstrated that overproduction of TNFR occurs chiefly outside the kidneys and suggests that leukocytes are one plausible non-kidney source of TNFR from elegant studies (36, 37).

The limitations of the study should be acknowledged. First, it is not clear whether long-term febuxostat treatment would lower serum TNFR levels, although those levels did not change after a relatively short-term (12 weeks) treatment. Second, our multivariate model may not be appropriately adjusted because inflammatory markers other than hsCRP are not measured. Finally, we use 30% decline in eGFR from baseline as alternative end point to ESRD or 57% decline in eGFR from baseline (doubling of serum creatinine level). Therefore, this endpoint may not accurately reflect future renal function decline. Nevertheless, the strength of the study is that baseline TNFR levels are shown to predict eGFR reduction despite limited reduction in eGFR in the study period.

## Conclusions

Elevated baseline TNFR levels were associated with eGFR decline; however, early change in circulating TNFR levels failed to predict future eGFR decline chiefly in non-diabetic CKD patients with asymptomatic hyperuricemia. Therefore, additional studies are required to identify treatments that modulate circulating TNFR levels.

## Data Availability Statement

The raw data supporting the conclusions of this article will be made available by the authors, without undue reservation.

## Ethics Statement

The studies involving human participants were reviewed and approved by the Institutional Review Board of the Juntendo University Faculty of Medicine, Tokyo, Japan (No. H18-0204). Written informed consent for participation was not required for this study in accordance with the national legislation and the institutional requirements.

## Author Contributions

TG and NY helped to design the study, oversee its execution, and write the manuscript. YO, IO, YSh, NI, SI, HH, KK, and TH helped to provide clinical data and write the manuscript. MM, YN, SN, SU, and YSu helped to interpret data. All authors contributed to the article and approved the submitted version.

## Conflict of Interest

The authors declare that the research was conducted in the absence of any commercial or financial relationships that could be construed as a potential conflict of interest.

## Abbreviations

ACR: albumin to creatinine ratio
ANCOVA: analysis of covariance
BMI: body mass index
BP: blood pressure
CI: confidence interval
CKD: chronic kidney disease
DKD: diabetic kidney disease
eGFR: estimated glomerular filtration rate
ELISA: enzyme-linked immunosorbent assay
FEATHER: Febuxostat vs. Placebo Randomized Controlled Trial Regarding Reduced Renal Function in Patients With Hyperuricemia Complicated by Chronic Kidney Disease Stage 3
HbA1c: glycated hemoglobin
hsCRP: high sensitivity C-reactive protein
ICAM-1: intercellular adhesion molecule-1
IL: interleukin
JAK: janus kinase
NLRP3: NOD, LRR, and pyrin domain-containing protein 3
PERL: Preventing Early Renal Loss in Diabetes
RCT: randomized controlled trial
SD: standard deviation
SGLT: sodium glucose cotransporter
TNF: tumor necrosis factor
TNFR: TNF receptor.

## References

[1] Iseki K Ikemiya Y Inoue T Iseki C Kinjo K Takishita S. Significance of hyperuricemia as a risk factor for developing ESRD in a screened cohort. Am J Kidney Dis. (2004) 44:642–50. 10.1016/S0272-6386(04)00934-5 15384015

[2] Tseng WC Chen YT Lin YP Ou SM Yang CY Lin CH. Hyperuricemia predicts an early decline in renal function among older people: a community-based cohort study. Sci Rep. (2019) 9:980. 10.1038/s41598-018-37529-z 30700753 PMC6353916

[3] Ficociello LH Rosolowsky ET Niewczas MA Maselli NJ Weinberg JM Aschengrau A. High-normal serum uric acid increases risk of early progressive renal function loss in type 1 diabetes: results of a 6-year follow-up. Diabetes Care. (2010) 33:1337–43. 10.2337/dc10-0227 20332356 PMC2875450

[4] Doria A Galecki AT Spino C Pop-Busui R Cherney DZ Lingvay I. Serum urate lowering with allopurinol and kidney function in type 1 diabetes. New Engl J Med. (2020) 382:2493–503. 10.1056/NEJMoa1916624 32579810 PMC7375708

[5] Badve SV Pascoe EM Tiku A Boudville N Brown FG Cass A. Effects of allopurinol on the progression of chronic kidney disease. New Engl J Med. (2020) 382:2504–13. 10.1056/NEJMoa1915833 32579811

[6] Kimura K Hosoya T Uchida S Inaba M Makino H Maruyama S. Febuxostat therapy for patients with stage 3 CKD and asymptomatic hyperuricemia: a randomized trial. Am J Kidney Dis. (2018) 72:798–810. 10.1053/j.ajkd.2018.06.028 30177485

[7] Lin TC Hung LY Chen YC Lo WC Lin CH Tam KW. Effects of febuxostat on renal function in patients with chronic kidney disease: a systematic review and meta-analysis. Medicine. (2019) 98:e16311. 10.1097/md.0000000000016311 31335677 PMC6709169

[8] Kang DH. Hyperuricemia and progression of chronic kidney disease: role of phenotype transition of renal tubular and endothelial cells. Contrib Nephrol. (2018) 192:48–55. 10.1159/000484278 29393109

[9] Jung SW Kim SM Kim YG Lee SH Moon JY. Uric acid and inflammation in kidney disease. Am J Physiol Renal Physiol. (2020) 18:F1327–40. 10.1152/ajprenal.00272.2019 32223310

[10] Gohda T Niewczas MA Ficociello LH Walker WH Skupien J Rosetti F. Circulating TNF receptors 1 and 2 predict stage 3 CKD in type 1 diabetes. J Am Soc Nephrol. (2012) 23:516–24. 10.1681/ASN.2011060628 22266664 PMC3294299

[11] Niewczas MA Gohda T Skupien J Smiles AM Walker WH Rosetti F. Circulating TNF receptors 1 and 2 predict ESRD in type 2 diabetes. J Am Soc Nephrol. (2012) 23:507–15. 10.1681/ASN.2011060627 22266663 PMC3294310

[12] Gohda T Maruyama S Kamei N Yamaguchi S Shibata T Murakoshi M. Circulating TNF receptors 1 and 2 predict mortality in patients with end-stage renal disease undergoing dialysis. Sci Rep. (2017) 7:43520. 10.1038/srep43520 28256549 PMC5335256

[13] Neirynck N Glorieux G Schepers E Verbeke F Vanholder R. Soluble tumor necrosis factor receptor 1 and 2 predict outcomes in advanced chronic kidney disease: a prospective cohort study. PLoS ONE. (2015) 10:e0122073. 10.1371/journal.pone.0122073 25823004 PMC4379033

[14] Heerspink HJL Perco P Mulder S Leierer J Hansen MK Heinzel A. Canagliflozin reduces inflammation and fibrosis biomarkers: a potential mechanism of action for beneficial effects of SGLT2 inhibitors in diabetic kidney disease. Diabetologia. (2019) 62:1154–66. 10.1007/s00125-019-4859-4 31001673 PMC6560022

[15] Niewczas MA Pavkov ME Skupien J Smiles A Md Dom ZI Wilson JM. A signature of circulating inflammatory proteins and development of end-stage renal disease in diabetes. Nat Med. (2019) 25:805–13. 10.1038/s41591-019-0415-5 31011203 PMC6508971

[16] Sonoda Y Gohda T Suzuki Y Omote K Ishizaka M Matsuoka J. Circulating TNF receptors 1 and 2 are associated with the severity of renal interstitial fibrosis in IgA nephropathy. PLoS ONE. (2015) 10:e0122212. 10.1371/journal.pone.0122212 25860248 PMC4393287

[17] Kamei N Yamashita M Nishizaki Y Yanagisawa N Nojiri S Tanaka K. Association between circulating tumor necrosis factor-related biomarkers and estimated glomerular filtration rate in type 2 diabetes. Sci Rep. (2018) 8:15302. 10.1038/s41598-018-33590-w 30333553 PMC6193030

[18] Murakoshi M Gohda T Sonoda Y Suzuki H Tomino Y Horikoshi S. Effect of tonsillectomy with steroid pulse therapy on circulating tumor necrosis factor receptors 1 and 2 in IgA nephropathy. Clin Exp Nephrol. (2017) 21:1068–74. 10.1007/s10157-017-1408-7 28389814

[19] Gohda T Nishizaki Y Murakoshi M Nojiri S Yanagisawa N Shibata T. Clinical predictive biomarkers for normoalbuminuric diabetic kidney disease. Diabetes Res Clin Pract. (2018) 141:62–8. 10.1016/j.diabres.2018.04.026 29729375

[20] Kang DH Park SK Lee IK Johnson RJ. Uric acid-induced C-reactive protein expression: implication on cell proliferation and nitric oxide production of human vascular cells. J Am Soc Nephrol. (2005) 16:3553–62. 10.1681/ASN.2005050572 16251237

[21] Liang WY Zhu XY Zhang JW Feng XR Wang YC Liu ML. Uric acid promotes chemokine and adhesion molecule production in vascular endothelium via nuclear factor-kappa B signaling. Nutr Metab Cardiovasc Dis. (2015) 25:187–94. 10.1016/j.numecd.2014.08.006 25315669

[22] Zhou Y Fang L Jiang L Wen P Cao H He W. Uric acid induces renal inflammation via activating tubular NF-kappaB signaling pathway. PLoS ONE. (2012) 7:e39738. 10.1371/journal.pone.0039738 22761883 PMC3382585

[23] Xiao J Fu C Zhang X Zhu D Chen W Lu Y. Soluble monosodium urate, but not its crystal, induces toll like receptor 4-dependent immune activation in renal mesangial cells. Mol Immunol. (2015) 66:310–8. 10.1016/j.molimm.2015.03.250 25909495

[24] Kim SM Lee SH Kim YG Kim SY Seo JW Choi YW. Hyperuricemia-induced NLRP3 activation of macrophages contributes to the progression of diabetic nephropathy. Am J Physiol Renal Physiol. (2015) 308:F993–1003. 10.1152/ajprenal.00637.2014 25651569

[25] Lee HJ Jeong KH Kim YG Moon JY Lee SH Ihm CG. Febuxostat ameliorates diabetic renal injury in a streptozotocin-induced diabetic rat model. Am J Nephrol. (2014) 40:56–63. 10.1159/000363421 25034030

[26] Zhou Y Zhao M Pu Z Xu G Li X. Relationship between oxidative stress and inflammation in hyperuricemia: analysis based on asymptomatic young patients with primary hyperuricemia. Medicine. (2018) 97:e13108. 10.1097/MD.0000000000013108 30544373 PMC6310523

[27] Myllymaki J Honkanen T Syrjanen J Helin H Rantala I Pasternack A. Uric acid correlates with the severity of histopathological parameters in IgA nephropathy. Nephrol Dial Transplant. (2005) 20:89–95. 10.1093/ndt/gfh584 15572382

[28] Kamijo-Ikemori A Sugaya T Hibi C Nakamura T Murase T Oikawa T. Renoprotective effect of the xanthine oxidoreductase inhibitor topiroxostat on adenine-induced renal injury. Am J Physiol Renal Physiol. (2016) 310:F1366–76. 10.1152/ajprenal.00517.2015 27029427

[29] Muir SW Harrow C Dawson J Lees KR Weir CJ Sattar N. Allopurinol use yields potentially beneficial effects on inflammatory indices in those with recent ischemic stroke: a randomized, double-blind, placebo-controlled trial. Stroke. (2008) 39:3303–7. 10.1161/STROKEAHA.108.519793 18845806

[30] Takir M Kostek O Ozkok A Elcioglu OC Bakan A Erek A. Lowering uric acid with allopurinol improves insulin resistance and systemic inflammation in asymptomatic hyperuricemia. J Investig Med. (2015) 63:924–9. 10.1097/JIM.0000000000000242 26571421

[31] Jalal DI Decker E Perrenoud L Nowak KL Bispham N Mehta T. Vascular function and uric acid-lowering in stage 3 CKD. J Am Soc Nephrol. (2017) 28:943–52. 10.1681/ASN.2016050521 27620990 PMC5328166

[32] Greenberg JH Abraham AG Xu Y Schelling JR Feldman HI Sabbisetti VS. Plasma biomarkers of tubular injury and inflammation are associated with CKD progression in children. J Am Soc Nephrol. (2020) 31:1067–77. 10.1681/ASN.2019070723 32234829 PMC7217410

[33] Bhatraju PK Zelnick LR Shlipak M Katz R Kestenbaum B. Association of soluble TNFR-1 concentrations with long-term decline in kidney function: the multi-ethnic study of atherosclerosis. J Am Soc Nephrol. (2018) 29:2713–21. 10.1681/ASN.2018070719 30287518 PMC6218870

[34] Mikami R Mizutani K Gohda T Gotoh H Matsuyama Y Aoyama N. Association between circulating tumor necrosis factor receptors and oral bacterium in patients receiving hemodialysis: a cross-sectional study. Clin Exp Nephrol. (2020) 25:58–65. 10.1007/s10157-020-01952-2 32816134

[35] Gohda T Kamei N Kubota M Tanaka K Yamashita Y Sakuma H. Fractional excretion of tumor necrosis factor receptor 1 and 2 in patients with type 2 diabetes and normal renal function. J Diabetes Investig. (2021) 12:382–9. 10.1111/jdi.13351 32643269 PMC7926211

[36] Wheelock KM Saulnier PJ Tanamas SK Vijayakumar P Weil EJ Looker HC. White blood cell fractions correlate with lesions of diabetic kidney disease and predict loss of kidney function in Type 2 diabetes. Nephrol Dial Transplant. (2017) 32:2145. 10.1093/ndt/gfx303 29088394

[37] Chow F Ozols E Nikolic-Paterson DJ Atkins RC Tesch GH. Macrophages in mouse type 2 diabetic nephropathy: correlation with diabetic state and progressive renal injury. Kidney Int. (2004) 65:116–28. 10.1111/j.1523-1755.2004.00367.x 14675042

